# Multimorbidity: the case for prevention

**DOI:** 10.1136/jech-2020-214301

**Published:** 2020-10-05

**Authors:** Anna Head, Kate Fleming, Chris Kypridemos, Jonathan Pearson-Stuttard, Martin O’Flaherty

**Affiliations:** 1 Department of Public Health, Policy and Systems, University of Liverpool, Liverpool, UK; 2 School of Public Health, Imperial College London, London, UK

**Keywords:** Public health, Prevention, Epidemiology of chronic diseases

## Abstract

Multimorbidity is of increasing concern for healthcare systems globally, particularly in the context of ageing population structures, such as in the European Union and the UK. Although there is growing attention on developing strategies to manage the health and healthcare burden of older patients with multimorbidity, little research or policy focus has been placed on how to best prevent the development of multimorbidity in future generations. In this research agenda piece, we argue for a shift from a sole focus on the management of multimorbidity in old age to a multimorbidity agenda that considers prevention and management throughout the life-course.

##  

Multimorbidity, commonly defined as the coexistence of two or more chronic conditions,^1^ is an increasingly critical priority for health systems and health research globally.^2 3^ In the UK, an estimated one in four patients in primary care have multiple chronic conditions,^4^ and in the USA, the prevalence is approximately 25% of the general adult population.^5^ Estimates of multimorbidity worldwide are similarly high across low-income, middle-income and high-income countries.^6^ Despite projections that the number of people living with multimorbidity will continue to increase,^7^ policy and research remain focused on single diseases. Multimorbidity, and particularly how to better prevent or postpone the accumulation of multiple diseases, has received much less attention.

The two leading drivers of multimorbidity are age and socioeconomic disadvantage.^6^ The prevalence of multimorbidity is higher, and age of onset is younger, among those living in more deprived areas.^4 8^ Several studies have found socioeconomic inequalities in all but the oldest age groups (80+/85+),^4 8 9^ with the inequalities gap appearing to be widest in the older working-age population and those just after retirement age.^4 9 10^ Furthermore, certain combinations of conditions are more common in more deprived groups.^4 8^ This includes multimorbidity involving mental health conditions and multimorbidity with 10 or more functional limitations, with those in the most deprived quintile having a 90% higher prevalence than those in the least deprived quintile.^11^ While socioeconomic inequalities are present in the transition from a healthy state to having multimorbidity, there is evidence they are not present in the transition from multimorbidity to mortality.^12 13^ In addition, material, psycho-social and behavioural determinants of health have all been associated with multimorbidity.^11 12^

Several studies have found that multimorbidity is becoming increasingly prevalent at younger ages. The Twenty-07 cohort study in Scotland reported a 60% increase in the prevalence of multimorbidity at age 60 in the cohort born in the 1950s compared with the cohort born in the 1930s.^10^ Analysis of the English Longitudinal Study of Ageing found that the age when the majority of participants reporting multimorbidity decreased from 70–74 between 2002–2011, to the 65–69 age group from 2012/2013 onwards.^9^ With increasing prevalence of risk factors such as obesity, particularly in childhood,^14^ and childhood socioeconomic conditions shown to be associated with morbidity and multimorbidity in later life,^11 15^ these life-course exposures may provide clues for these trends towards younger onset of multimorbidity.

Multimorbidity has been described as a ‘defining challenge’ for health systems,^16^ which are traditionally focused around single conditions.^3^ People with multimorbidity are estimated to make up approximately half of primary care consultations in England and 75% of prescriptions.^4^ Additional consequences of multimorbidity such as frailty and functional decline heavily impact an individual’s independence and increase strain upon the social care system.^7^ This is a major policy concern for the next decade,^17^ compounded in many countries by ageing population structures. Furthermore, poor health and functional impairment are associated with earlier exit from work.^18^ There are also wider economic impacts with the total production losses of all-cause premature mortality and morbidity in the 28 European countries estimated at €175 billion, equivalent to an economic burden of 1.2% of the gross domestic product.^19^

There is increasing awareness of the need to manage this growing burden of multimorbidity.^2 3^ To date, discussions have primarily been around reducing the risk in older patients of adverse events such as unplanned admissions or reducing the cost of primary care management in complex patients.^20^ Alongside reducing the impacts of multimorbidity, a slow down in incidence is needed in order to substantially decrease its overall health burden, reduce the pressure on health and social care systems and allow patients to remain in better health for longer. Attention, therefore, also needs to be paid to identifying the most effective ways of preventing or postponing the onset of multimorbidity.^2^

This research agenda for multimorbidity prevention should identify effective and equitable strategies for reducing risk factor exposure levels for common disease combinations, consider the optimum balance between strategies for the prevention of initial disease onset and the onset of subsequent diseases, and predict the potential of different interventions to delay and/or prevent the onset of multimorbidity. Such research requires (1) a longitudinal perspective focused on drivers of inequalities, (2) an integrated approach across the life-course and care systems that address the social determinants of health and (3) inclusive definitions of multimorbidity.

Causal associations between amenable risk factors and non-communicable diseases (NCDs), a large proportion of the multimorbidity burden,^6^ are well established. Approximately one-third of disability-adjusted life years from NCDs in 2017 are estimated to be attributed to metabolic and behavioural risk factors such as smoking, body mass index and blood pressure.^21^ We now have many evidence-based individual-level and population-level effective prevention strategies for individual NCDs.^22^ As NCD risk factors accumulate over the life-course, integrated prevention strategies can and should be used to intervene at all ages and stages of disease progression through both primary care and secondary care. We currently know little about the independent risk factors or the accumulation trajectories for multimorbidity, so prospective studies looking at long-term trends in how NCD prevention impacts multimorbidity incidence are needed.

Traditionally, evaluations of prevention strategies estimate the effectiveness of interventions in improving individual disease outcomes. The shared aetiology of many NCDs means that reducing risk factor exposure may have exponential payoff by reducing the incidence of multiple conditions. Explicitly including multimorbidity as a health outcome in evaluating the effectiveness and equity of prevention strategies will help to identify potential additional benefits of interventions. Developments in microsimulation modelling and survival analysis methods for multivariate failure time data can also be instrumental in identifying risk factors for common disease combinations.

It is important that prevention strategies are equitable as well as effective.^23 24^ In particular, individual-level behaviour change interventions alone may increase socioeconomic inequalities in health as they require substantial agency for individuals and providers to implement solutions effectively.^24^ As mentioned earlier, there is also strong evidence of the cumulative effects of early experiences on health outcomes in later life.^25^ The case for population-level, structural policies to decrease incidence and premature mortality associated with many NCDs has been shown through empirical evaluations and modelling studies comparing prevention interventions in policy domains such as tobacco, food systems and the built environment. These population-level policies also have greater potential to reduce socioeconomic inequalities than individual-level behaviour change interventions.^24 26^

No one single definition allows for the exploration of all issues related to multimorbidity. From a population-level policy perspective, it is necessary to estimate the extent of both preventable multimorbidity and non-preventable multimorbidity. This requires researchers to operationalise multimorbidity definitions in a way that is general and relevant to patients of all ages, clinicians and healthcare systems.^2^ This should include a broad scope of conditions covering not only physical NCDs associated with ageing, but also mental health conditions, chronic infectious diseases and conditions that are risk factors for other diseases. Researchers should thus be transparent with justification for included or excluded conditions, disease lists and coding algorithms to enable replication and comparison.

The importance of structural drivers, and crucially social determinants, in driving the NCD burden and multimorbidity means preventative efforts are not limited to the health and social care sectors.^25^ Regulatory, fiscal and policy strategies to shape the various environments that generate disease will need intersectoral and whole-of-government approaches, with a strong local focus and inclusion of civil society.^27^ A prevention approach to multimorbidity ties into both the healthy ageing agenda of increasing years of life lived in good health^28^ and the WHO’s call for a systems approach to multimorbidity strongly focused on the social determinants of health.^1^ Furthermore, a multimorbidity prevention agenda aligns well with the third UN Sustainable Development Goal (SDG) of ensuring healthy lives and promoting well-being for all at all ages.^29^ Strategies to prevent multimorbidity can contribute to SDG target 3.4 of reducing premature mortality from NCDs by one-third between 2015 and 2030. Overall, a social determinant of health approach to addressing inequalities underlies these agendas for reducing NCDs and multimorbidity, and for promoting healthy ageing.

We need to challenge the common narrative that multimorbidity is inevitable in a modern ageing society. To do this, the focus on multimorbidity must shift from solely management of high-risk older individuals to include integrated population-level prevention strategies throughout the life-course to address the drivers of multimorbidity. Achieving compression of amenable morbidity is essential for the sustainability of future healthcare and social care systems and for tackling the gap in health outcomes and socioeconomic inequalities more broadly.

**Key messages**

- Multimorbidity is of growing concern globally, due to its increasing prevalence, its unequal social distribution, and its impact on health outcomes as well as health and social care systems.
- Policy and research remain primarily focused on single diseases rather than multimorbidity, and how to better prevent the accumulation of multiple diseases has received even less attention.
- Multimorbidity is not just a problem of managing complex conditions in older age. Non-communicable diseases with amenable risk factors form a large proportion of the multimorbidity burden, providing opportunities throughout the life-course for population health prevention strategies.
